# The *Tgif2 *gene contains a retained intron within the coding sequence

**DOI:** 10.1186/1471-2199-7-2

**Published:** 2006-01-25

**Authors:** Tiffany A Melhuish, David Wotton

**Affiliations:** 1 Department of Biochemistry and Molecular Genetics, Center for Cell Signaling, University of Virginia, Hospital West, Box 800577, HSC, Charlottesville VA 22908, USA

## Abstract

**Background:**

TGIF and TGIF2 are homeodomain proteins, which act as TGFβ specific Smad transcriptional corepressors. TGIF recruits general repressors including mSin3 and CtBP. The related TGIF2 protein functions in a similar manner, but does not bind CtBP. In addition to repressing TGFβ activated gene expression, TGIF and TGIF2 repress gene expression by binding directly to DNA. TGIF and TGIF2 share two major blocks of similarity, encompassing the homeodomain, and a conserved carboxyl terminal repression domain. Here we characterize two splice variants of the Tgif2 gene from mouse and demonstrate that the Tgif2 gene contains a retained intron.

**Results:**

By PCR from mouse cDNA, we identified two alternate splice forms of the Tgif2 gene. One splice variant encodes the full length 237 amino acid Tgif2, whereas the shorter form results in the removal of 39 codons from the centre of the coding region. The generation of this alternate splice form occurs with the mouse RNA, but not the human, and both splice forms are present in all mouse tissues analyzed. Human and mouse Tgif2 coding sequences contain a retained intron, which in mouse Tgif2 is removed by splicing from around 25–50% of RNAs, as assessed by RT-PCR. This splicing event is dependent on sequences within the mouse Tgif2 coding sequence. Both splice forms of mouse Tgif2 encode proteins which are active transcriptional repressors, and can repress both TGFβ dependent and independent transcription. In addition, we show that human and mouse Tgif2 interact with the transcriptional corepressor mSin3.

**Conclusion:**

These data demonstrate that the Tgif2 gene contains a retained intron, within the second coding exon. This retained intron is not removed from the human mRNA at a detectable level, but is spliced out in a significant proportion of mouse RNAs. This alternate splicing is dependent entirely on sequences within the mouse Tgif2 coding sequence, suggesting the presence of an exonic splicing enhancer. Both splice forms of mouse Tgif2 produce proteins which are functional transcriptional repressors.

## Background

TGIF (thymine guanine interacting factor) is a homeodomain containing protein which was first identified by its ability to bind adjacent to a specific retinoic acid response element [1]. TGIF is a transcriptional repressor, which interacts with general corepressors, including CtBP and the mSin3A/histone deacetylase (HDAC) complex [2-5]. Loss of function mutations in human TGIF are associated with holoprosencephaly (HPE), a severe genetic disease affecting craniofacial development [6-8]. A human TGIF related protein (TGIF2) has been identified, which shares at least some functions with TGIF [9,10]. TGIF and TGIF2 are also transcriptional corepressors for TGFβ receptor activated Smads [9,11]. TGIF interacts specifically with Smad2 and Smad3, and is thought to limit the magnitude of the transcriptional response to TGFβ signaling. In response to the binding of TGFβ to its cognate receptors, the activated receptor complex phosphorylates and activates specific receptor Smad (R-Smad) proteins: Smad2 or Smad3 in the case of TGFβ and activin [12-14]. The activated R-Smads then complex with the co-Smad, Smad4 and accumulate in the nucleus, where they activate expression of specific target genes. The Smad proteins themselves can bind to DNA, and are also recruited to specific response elements via interactions with other sequence specific DNA binding proteins [15-20]. Once bound to a specific target DNA element, a Smad complex activates transcription via interactions with general coactivators, such as p300/CBP [21-24]. In addition to coactivator interactions, Smads can also interact with specific transcriptional corepressors, including TGIF and TGIF2 [9,11], c-Ski and the related SnoN [25-28]. These interactions result in a loss of activation due to displacement of the coactivator complex, and active repression of gene expression by the recruitment of general corepressors [11,17,29]. It is likely that the relative abundance of Smad coactivators and corepressors within a cell is a major determinant of the magnitude of the transcriptional response to TGFβ.

Since human TGIF and TGIF2 appear to be relatively divergent in primary amino acid sequence outside the two main blocks of homology [9,30], we wanted to identify and characterize TGIF2 from another species. Here we report the cloning and characterization of mouse Tgif2, as well as the identification of an unusual splice variant of the mouse Tgif2 gene. Analysis of this splice variant suggests that the TGIF2 genes from both human and mouse contain a retained intron, which is spliced out of a proportion of mouse Tgif2 RNAs.

## Results

### Two splice forms of mouse Tgif2

**Figure 1:**
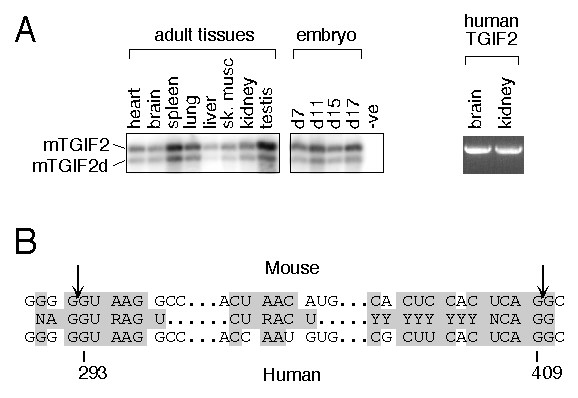
**Two splice forms of mouse Tgif2**. A) Expression of mouse Tgif2 was analyzed by PCR from 1^st ^strand cDNA, using primers at the 5' and 3' ends of the coding sequence. cDNAs were from the tissues indicated (sk. musc = skeletal muscle), or from embryos at the indicated days of gestation. -ve: no cDNA. Similar PCR analysis was carried out on cDNA from human brain and kidney (right). B) Alignment of putative splice sequences from mouse and human TGIF2 with splice consensus sequences (matches to the consensus are shaded gray).

**Figure 2:**
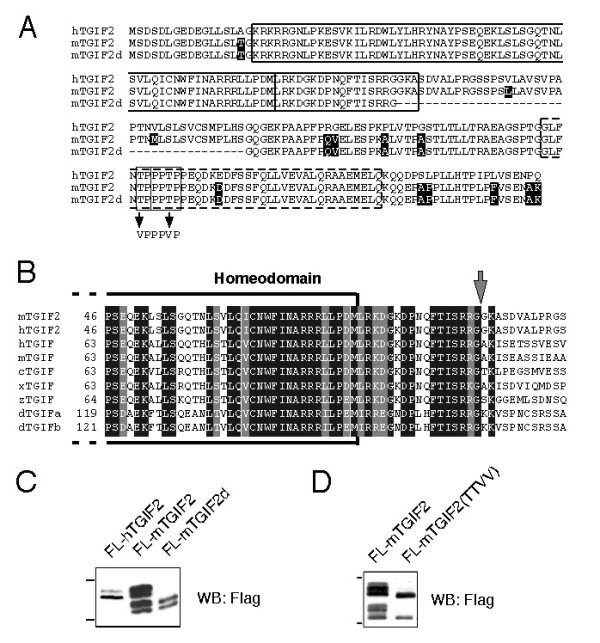
**Multiple forms of the mouse TGIF2 protein**. A) alignment of human TGIF2 and mouse Tgif2 and Tgif2d protein sequence. Differences between human and mouse are shaded. Dashes indicate amino acids missing in mTGIF2d. The homeodomain and carboxyl-terminal extension are boxed. The carboxyl-terminal region of TGIF2 which is conserved in TGIF is indicated by a dashed box. MAP kinase phosphorylation sites are boxed, and threonine to valine mutations are indicated below. B) Alignment of the carboxyl-terminal part of the homeodomain and extension from TGIFs of different species. TGIF2: human, mouse; TGIF: human, mouse, chicken, Xenopus, zebra fish; *Drosophila *dTGIFa and dTGIFb. Residues identical between all are shaded black, similar are gray. The homeodomain is boxed and the arrow indicates the position affected by alternate splicing of *mTgif2*. COS-1 cells were transfected with the indicated Flag-tagged expression constructs (C) or with Flag-mTGIF2 or a mutant in which two threonines were altered to valine (D). Protein expression was analyzed by western blotting with a Flag specific antibody.

We isolated clones for mouse Tgif2 by PCR from 11 day mouse embryo cDNA using primers at the extreme 5' and 3' ends of the coding sequence. A band of the expected size for Tgif2 was amplified from this cDNA, and a faster migrating band was also present (Figure 1A and data not shown). Each of these DNA fragments was cloned and sequenced: The slower migrating form represented a mouse Tgif2 clone encoding a predicted 237 amino acid protein with only 13 amino acid differences from the human protein (see Figure 2). Interestingly, the faster migrating DNA fragment also contained a form of mouse Tgif2, but was lacking 117 base pairs from the centre of the cDNA. Inspection of the sequences that are missing in this clone revealed the presence of 5' and 3' splice sites as well as a 5/6 match to the branch point consensus sequence [31], suggesting that this deletion is caused by alternate splicing (Figure 1B). The branch point sequence is less well conserved (3/6 match) in the human TGIF2 gene, and we have been unable to amplify a similar form of human TGIF2 from cDNA (Figure 1A and data not shown). Thus, it appears that this alternately spliced form of Tgif2 (which we term Tgif2d) is not found to a significant degree in humans.

To determine how wide-spread the expression of the two splice forms of *mTgif2 *is, we PCR amplified mTgif2 cDNAs from a panel of mouse tissues. In all adult tissues where *mTgif2 *was detected, both forms of the mRNA were present, and both splice forms were present from day 7 of embryonic development (Figure 1A). In all tissues tested, the *mTgif2d *splice form was readily detectable by this RT-PCR assay. However, we have not carried out accurate quantification of the relative amounts of each splice form in multiple tissues.

### Both splice forms of Mouse Tgif2d produce proteins

The predicted proteins from the human and mouse *Tgif2 *genes are highly conserved, with 94% identity over the entire protein (Figure 2A). For comparison, TGIFs from these species are 89% identical. There are two regions of conservation between TGIF and TGIF2: The homeodomain plus a 20 amino acid carboxyl-terminal extension which is characteristic of TGIFs (Figure 2A, boxed), and a region near the carboxyl-termini of the proteins (Figure 2A, dashed box). As shown in Figure 2A, alternate splicing of *mTgif2 *removes part of the central linker joining the two regions of conservation between TGIF and TGIF2 proteins. We previously characterized two TGIF related proteins from *Drosophila*, that have a high degree of identity to vertebrate TGIFs in the homeodomain and the carboxyl-terminal extension [30], which is conserved in vertebrate TGIFs (Figure 2B). Interestingly, alternate splicing of *mTgif2 *retains the homeodomain and almost all of the conserved region carboxyl terminal to it, suggesting that the homeodomain plus carboxyl-terminal extension (HD+20) is not disrupted in mTgif2d.

To test whether the alternate splice form of *mTgif2 *is expressed as a stable protein product, we transfected COS-1 cells with Flag-tagged versions of human and mouse TGIF2 and mTgif2d. The human TGIF2 protein migrates as two, or three bands (the slowest migrating band is often seen with higher levels of expression) of apparent molecular weight of 30–32 kD (Figure 2C). This results from phosphorylation of two conserved MAP kinase sites within the carboxyl terminal region of the protein [9]. In cells expressing mTgif2, three bands in the 30–32 kD range were present, as well as a group of three bands with faster mobility (Figure 2C). The difference in mobility between these two triplets is consistent with the removal of approximately 4 kD predicted by removal of 39 codons from the centre of the coding sequence. Expression of the mTgif2d isoform resulted in the presence only of the lower group of bands. Alteration of the two threonine residues within the conserved MAP kinase sites to valines within mTgif2 (see Figure 2A) resulted in the presence of only two bands, suggesting that as with the human TGIF2 [9] the upper bands are due to phosphorylation of these residues (Figure 2D). Thus, it appears that multiple forms of mouse Tgif2 are created both by alternate splicing and phosphorylation.

### Mouse *Tgif2d *encodes a functional repressor which interacts with mSin3

**Figure 3:**
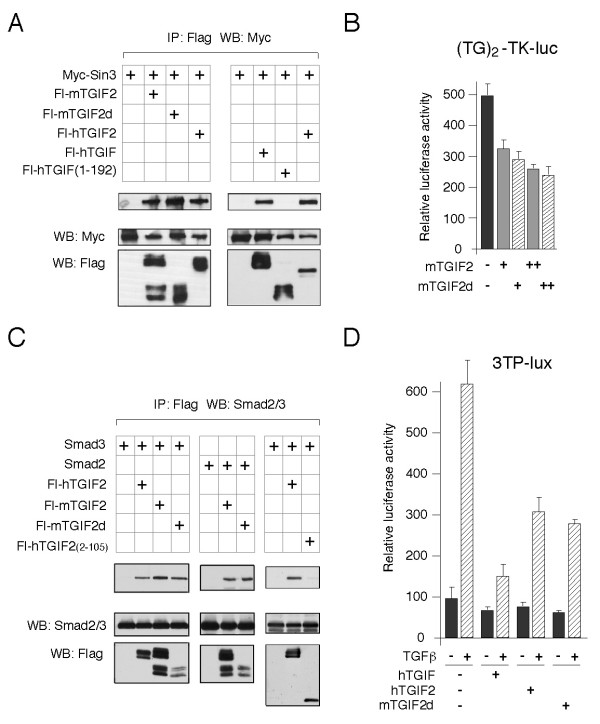
**The mouse Tgif2d protein is a functional repressor**. A) COS-1 cells were cotransfected with myc-tagged mSin3A and the indicated Flag-tagged expression constructs. Proteins were isolated on Flag agarose and analyzed for coprecipitating mSin3A with a myc antibody. Expression of transfected proteins in cell lysates is shown below. B) HepG2 cells were transfected with a reporter in which the luciferase gene is driven by the TK promoter and two TGIF binding sites, together with increasing amounts of mTgif2 or mTgif2d. Luciferase activity was assayed after 36 hours and is shown compared to a control transfection (mean +/- s.d. of triplicate transfections). C) COS-1 cells were cotransfected with a Smad2 or Smad3 expression vector and the indicated Flag-tagged expression constructs. Proteins were isolated on Flag agarose and analyzed for coprecipitating Smad2 or Smad3. Expression of transfected proteins in cell lysates is shown below. D) HepG2 cells were transfected with the 3TP-lux reporter, together with human TGIF, TGIF2 or mouse Tgif2d. After 24 hours, cells were treated with TGFβ (100 pM), or left untreated and luciferase activity was assayed 16 hours later, and is shown as the mean +/- s.d. of triplicate transfections.

To determine whether the mTgif2d protein functions in a similar manner to the longer splice form, we first looked at interactions with the general corepressor, mSin3. TGIF interacts with the corepressor mSin3 via its carboxyl terminal repression domain, which is conserved in human and mouse TGIF2 [4]. However, interaction of mSin3 with Tgif2 from mouse or human has not been demonstrated. COS-1 cells were transfected with expression constructs encoding Flag-tagged human or mouse TGIF2, or Flag-mTgif2d and Myc epitope-tagged mSin3. Protein complexes were isolated on Flag agarose and analyzed for the presence of Myc-Sin3. Both human and mouse TGIF2 interacted with mSin3, as did the mTgif2d splice variant, whereas, no interaction was seen with a carboxyl-terminal truncation mutant of hTGIF (Figure 3A). To test whether mTgif2d can repress transcription, we expressed either mTgif2 or mTgif2d in HepG2 cells and monitored expression of a luciferase reporter gene in which expression is driven by the TK promoter and two copies of a TGIF binding site (CTGTCAA). As shown in Figure 3B, both the full length mTgif2 and mTgif2d repressed expression of this reporter, suggesting that both splice forms are transcriptional repressors.

Both TGIF and human TGIF2 interact with TGFβ responsive Smads and repress transcription from TGFβ responsive reporters [9,11]. To determine whether alternate splicing of mouse *Tgif2 *affected interaction with TGFβ activated Smads, COS-1 cells were cotransfected with Smad2 or Smad3 expression plasmids together with human or mouse TGIF2. Smad3 clearly coprecipitated with both full length human and mouse TGIF2 and with the mTgif2d splice variant (Figure 3C). Similarly, Smad2 interacted with both forms of mouse Tgif2. However, we observed no interaction of Smad3 with a truncation mutant encoding amino acids 2–105 of hTGIF2 (Figure 3C). Together, these results suggest that sequences carboxyl-terminal to the homeodomain of TGIF2 are required for interaction with Smad3, and that the central region of the TGIF2 protein is dispensable for interaction with TGFβ activated Smads. To test whether the interaction of mTgif2d with Smad2 and Smad3 resulted in repression of TGFβ transcriptional responses, we cotransfected HepG2 cells with the 3TP-lux reporter and expression vectors encoding either human TGIF or TGIF2, or mouse Tgif2d. As shown in Figure 3D, all three proteins repressed the expression of this reporter in the presence of added TGFβ, although TGIF appeared to repress more effectively. These results suggest that both full length and the shorter isoform of mouse Tgif2 can act as Smad transcriptional corepressors.

### A retained intron in the second coding exon of Tgif2

**Figure 4:**
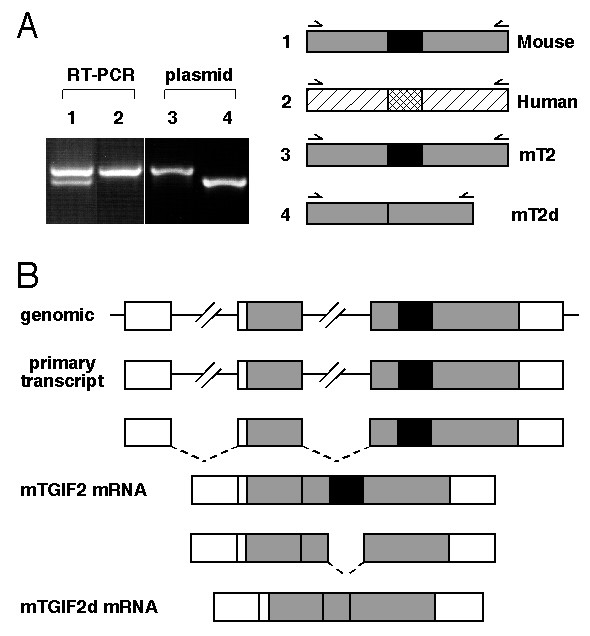
**A retained intron in the TGIF2 gene**. A) RT-PCR analysis of COS-1 cells transfected with mouse or human TGIF2 (lanes 1 and 2) or direct PCR on plasmid DNA (lanes 3 and 4). Positions of the PCR primers are shown to the right. B) A schematic representation of splicing events of the mouse Tgif2 RNA is shown.

To compare expression of human and mouse *Tgif2 *mRNAs in transfected cells, we generated expression vectors containing only the 237 amino acid ORFs from either species. As shown in Figure 4A (lane 1), RT-PCR analysis of RNA from COS-1 cells transiently transfected with a *mTgif2 *expression construct revealed the presence of both *mTgif2 *splice forms. In contrast, similar analysis of COS-1 cells transfected with the human *TGIF2 *ORF revealed only a single band corresponding to the full length 237 codon ORF (lane 2). For comparison, we also performed PCR reactions directly on plasmids containing the long and short forms of *mTgif2 *and obtained a single band of the expected size for each (Figure 4A, lanes 3 and 4). This suggests that alternate splicing of *Tgif2 *is specific to the mouse gene and independent of cell type. Additionally, it appears that the splicing event is dependent on the nucleotide sequence of the *mTgif2 *coding region. These data suggest that the mouse *Tgif2 *transcript can undergo multiple splicing events: Removal of the first and second introns from all RNAs, and removal of part of the second coding exon from a proportion of *mTgif2 *RNAs (Figure 4B). Thus the second coding exon of *mTgif2 *appears to contain a small intron, which is retained in at least half the mRNAs. In contrast, in the human mRNA, this intron is always retained, or removed at an extremely low frequency, below our level of detection.

### Analysis of the sequences required for alternate splicing

**Figure 5:**
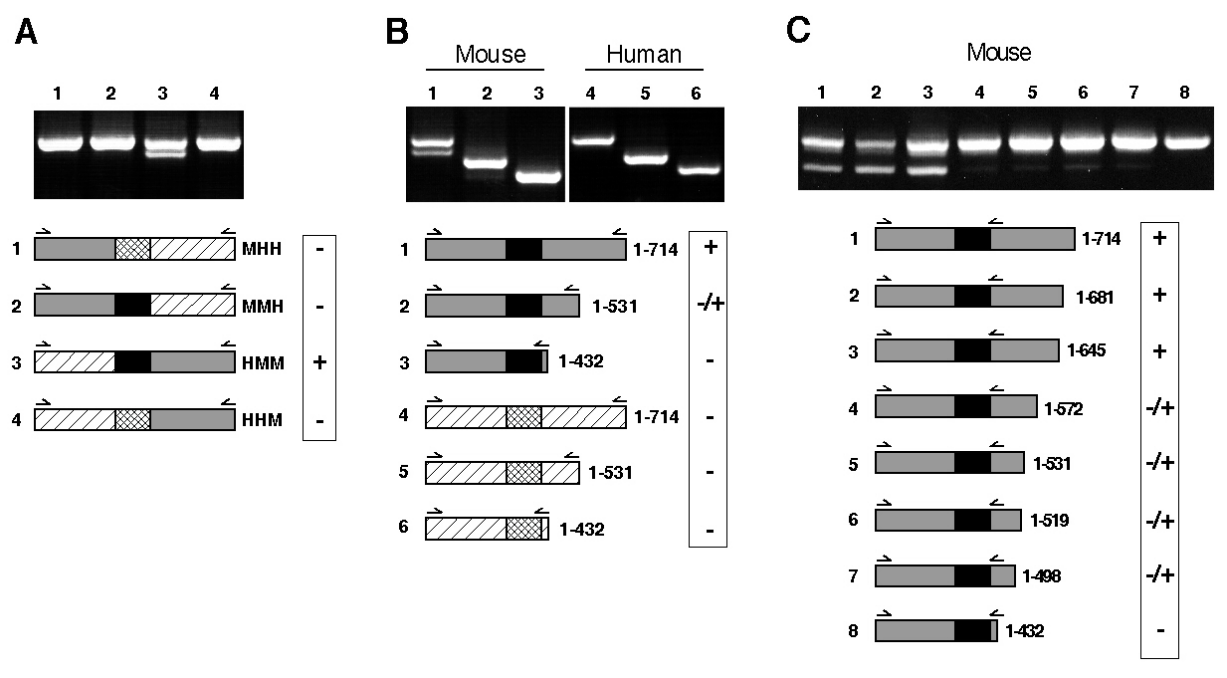
**The 3' coding region of mouse Tgif2 is required for alternate splicing**. COS-1 cells were transfected with expression constructs and expression of human, mouse and chimeric TGIF2 RNAs was analyzed by RT-PCR. The positions of primers are indicated (arrows). Alternate splicing: + splicing, - no splicing, -/+ just detectable, +/- slightly reduced. Constructs are shown schematically: Gray = mouse, black = retained intron. Stripes = human, crosshatch = retained intron. A) Comparison of constructs in which regions 5' and 3' of the retained intron are swapped between human and mouse TGIF2. B) Comparison of splicing of 3' deletion constructs for human and mouse TGIF2. C) Analysis of a series of 3' deletions of mouse TGIF2.

The previous data suggest that the *mTgif2 *coding region contains sequence information which directs alternate splicing, and this information is not present in the human. To determine which region of the *mTgif2 *RNA is required for alternate splicing, we first made a series of swaps between human and mouse *Tgif2*. The coding region was divided into three segments: The retained intron and the regions 5' and 3' to it. COS-1 cells were transfected with expression vectors containing these chimeric cDNAs and mRNAs were analyzed by RT-PCR. As shown in Figure 5A, both the retained intron and the region 3' of it from the mouse gene were required for alternate splicing (construct HMM). In contrast, constructs with either the 3' region alone (HHM), or both the retained intron and the 5' region (MMH) from the mouse did not splice out the retained intron. To determine whether sequences within the 3' region were inhibitory in the human gene or promoted splicing in the mouse gene, we tested two deletion constructs each for the human and mouse cDNAs. Neither of the human deletion constructs underwent alternate splicing, and the mouse 1–432 construct produced only a single splice form (Figure 5B). The intermediate deletion (1–531) resulted in a very low level of the alternate splice form, suggesting that the 3' coding region of the mouse gene contains sequences which enhance splicing. Analysis of a larger series of *mTgif2 *3' deletion constructs revealed that sequences 3' of base 645 were not required for alternate splicing (Figure 5C), whereas further truncation to base 572 dramatically reduced alternate splicing.

**Figure 6:**
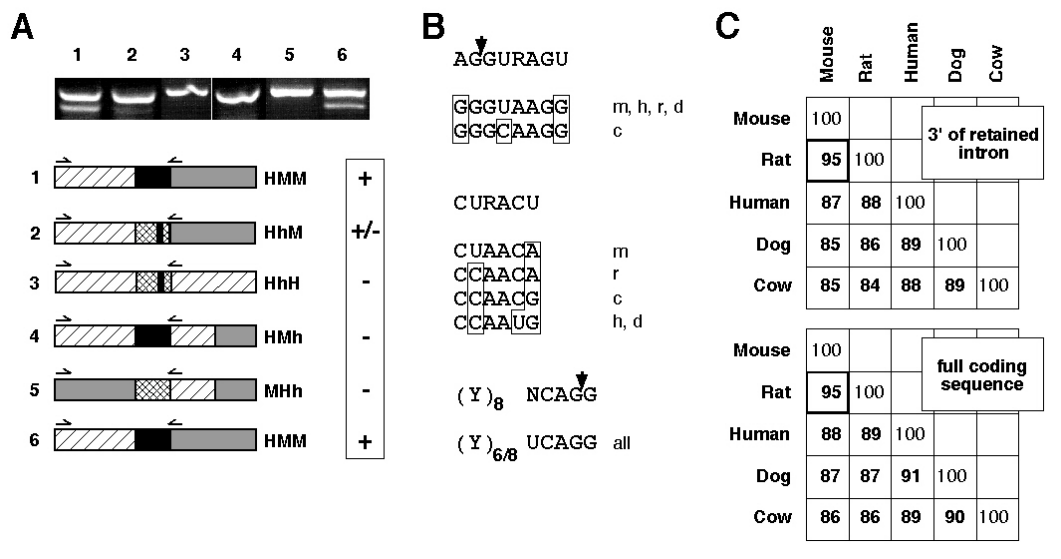
**The branch site sequence contributes to splicing efficiency**. A) Chimeric forms of Tgif2 were analyzed for splicing of the retained intron. A mutation of the branch site was introduced into the human TGIF2 sequence. HhH: the sequence of the branch acceptor site in the human was altered to that of mouse, HhM: as HhH, with mouse sequence 3' to the retained intron. HMh and MHh: the region 3' of the retained intron consisted of human sequences to base 543 and mouse sequence thereafter. The other regions of the coding sequence were human (H) or mouse (M) as indicated. B) Comparison of the sequences of the splice sites surrounding the retained intron and the branch acceptor site. Sequences from mouse (m), human (h), rat (r), dog (d) and cow (c) are shown with the consensus above. Boxed bases differ from the consensus. C) Pairwise sequence comparison of the coding sequence 3' of the retained intron (upper) or the entire coding sequences (lower) between the five species indicated. Percent identity is shown.

Mouse and human *Tgif2 *share a high degree of sequence identity in the 3' region of the coding sequence (89% identity over the entire coding region). We were, therefore, interested to determine whether we could transfer small regions of the mouse coding sequence to the human *Tgif2 *clone and cause it to undergo alternate splicing. We first altered the sequence surrounding the branch point in the retained intron from a 3/6 match to a 5/6 match to the consensus, as in the mouse sequence. As shown in Figure 6A, this change alone did not cause the human sequence to undergo alternate splicing (lane 3). When this alteration was combined with the mouse 3' region an intermediate level of splicing was observed (compare lanes 1, 2 and 3, Figure 6A). Thus, it appears that the branch point sequence plays a role in determining the differential splicing between human and mouse *Tgif2*. However, we cannot rule out contributions of other sequence differences within the retained intron.

To determine whether there was a simple sequence signal in the region of the mouse RNA 3' of the retained intron, we created two constructs in which mouse sequences 3' of base 543 were combined with the mouse retained intron (construct HMh) or the mouse 5' region (construct MHh). Within the chimeric 3' region, bases 410–543 come from the human and 544–714 from the mouse. This leaves only 16 base differences between the mouse sequence and the HMh construct over the retained intron and 3' region combined. When transfected into COS-1 cells, neither of these constructs underwent alternate splicing to any detectable level (Figure 6A, lanes 4 and 5). This suggests that if there is a splicing enhancer sequence in the 3' of the mouse coding sequence it is either relatively large or composed of multiple elements throughout the region. Together, these results suggest that the mouse *Tgif2 *coding sequence contains multiple positively acting signals which increase the efficiency of alternate splicing to remove a retained intron. To further explore the species specificity of the alternate splicing of Tgif2, we performed RT-PCR analyses on cell lines from different mammalian species, and searched available sequence databases. Interestingly, Tgif2d splice forms are present in the database from both mouse and rat, but not for any other vertebrate species for which Tgif2 clones are present. Consistent with the results from database searching, we have been unable to amplify by RT-PCR, or PCR from cDNA libraries a human or dog Tgif2d splice variant. Thus, it appears that the Tgif2d splice variant may be specific to rodents.

## Discussion

We show that the mouse Tgif2 gene undergoes alternative splicing, whereas, the human TGIF2 gene appears not to generate the alternate splice form at any significant level. Interestingly the alternate splicing seen in the mouse Tgif2 gene is dependent on the coding sequence of the mouse Tgif2 mRNA. Alternate splicing of the isolated mouse coding sequence occurs in all cell lines tested, and we did not detect significant levels of splicing of equivalent human TGIF2 constructs. Importantly, the pattern of splicing of the isolated human and mouse coding regions appears to correlate with that of the endogenous genes. Alternate splicing of the mouse RNA results in the removal of a region of the second coding exon, without disrupting the reading frame. At least half the time, this region is retained in the mouse RNA and appears to be always retained in the human. Thus it is possible that the human TGIF2 gene contains a constitutively retained intron. Searching the sequence databases for TGIF2 genes from other species revealed full length coding sequences from rat, cow and dog. As shown in Figure 5B, 5' and 3' splice sites did not differ in four of the five species, and only one difference was present in the cow sequence. However, the branch acceptor site in the mouse sequence was closer to the consensus than any of the other species and human and dog were most different, with 3/6 mismatches. Pairwise comparison of the sequences 3' to the retained intron between these five species revealed a high degree of identity (at least 84%), which is very similar to that for the entire coding sequence (Figure 5C). However, the mouse and rat sequences share 95% identity, whereas all other sequences are less than 90% identical.

Alternate splicing is likely to be the result of competition for spliceosome assembly between multiple splice sites, whereas, for intron retention it may be that the competition is between spliceosome assembly and RNA export [32]. In the human TGIF2 RNA, spliceosome assembly is too inefficient at these sites, whereas in the mouse RNA it occurs with a high enough efficiency that the retained intron is removed from a significant proportion of RNAs. Although this type of alternate splicing is relatively uncommon in animal cells, compared to the variable use of alternate splice sites [32,33], database analysis suggests that up to 14% of human genes have intron retention events. However, unlike the case reported here, the majority of these were found to be in untranslated regions [33].

We were unable to define a small region within the mouse Tgif2 coding sequence which promoted splicing, but it appears that the splicing event is dependent on much of the second coding exon. In many cases, alternate or regulated splicing is dependent on the presence of splicing enhancers, which can be located within exons. Splicing enhancer consensus sequences have been identified, and shown to bind to SR proteins such as SF2/ASF, SRp40 and SRp55 [34-37]. Binding of SR proteins to splicing enhancers is thought to facilitate the recruitment of the splicing machinery to adjacent introns, which can be several hundred bases distant. Although some matches to splicing enhancer sequences are present in the mouse Tgif2 coding sequence, we were unable to identify one which was responsible for the difference between human and mouse splicing. Comparison of the human and mouse Tgif2 sequences in the region 3' to the retained intron did not reveal the presence of a splicing enhancer consensus that was present in the mouse but disrupted by sequence differences in the human. Analysis of the coding sequences of human and mouse Tgif2 reveals the presence of a large number of predicted exonic splicing enhancer (ESE) consensus sequences (using both ESEFinder; [38] and RESCUE-ESE; [39]). We were unable to identify clear candidate ESE sequences which were present only in the mouse and not in the human, or which had much higher scores in the mouse than the human sequence. This may fit with our results which suggest that multiple regions of the mouse coding sequence promote alternate splicing.

## Conclusion

The Tgif2 gene contains a retained intron within the second coding exon. In the mouse, this retained intron is removed by splicing in a significant proportion of mRNAs, whereas, we have been unable to detect this splice form of the human TGIF2. It appears that the mouse Tgif2 coding region 3' of the retained intron contains sequences which promote removal of the retained intron in a cell type independent manner. In addition, sequences within the retained intron also contribute to the efficiency of splicing.

## Methods

### Cell culture and transfection

HepG2 and COS-1 cells were grown in Dulbecco's modified essential medium with high glucose and 10% fetal bovine serum. HepG2 cells were transfected in twelve-well plates using Exgen 500 (Fermentas) and COS-1 were transfected using LipofectAMINE (Invitrogen).

### Plasmids

3TP-lux contains a TGFβ-inducible promoter region from the *PAI-1 *gene and three TPA-response elements [40]. The (TG)2-TK-luc reporter is as described [5]. TGIF was expressed from a modified pCMV5 plasmid, with a Flag tag. Mouse Tgif2 constructs were created in pCMV5-Flag by PCR. Flag tagged human TGIF2 has been described [9]. Full length human Smad2 and Smad3 were expressed from pCMV5 without epitope tags and Myc-Sin3 and hTGIF(1–192) are as described [4]. Point mutations in mTgif2 were introduced by PCR, and verified by sequence analysis. hTGIF2(2–105) was created within pCMV5-Flag by PCR.

### Cloning of mouse Tgif2 cDNAs

Mouse Tgif2 was cloned by PCR from day 11 mouse embryo cDNA (Clontech). Primers, corresponding to the 5' and 3' ends of the expected coding sequence were based on the human sequence and the partial sequence of an EST clone, with a 5' EcoRI site and a 3' BamHI site. PCR products were analyzed by agarose gel electrophoresis and the two visible bands (approximately 600 bp and 700 bp) were excised from the gel, digested with EcoRI and BamHI and ligated into pBSKS- (Stratagene). Three individual clones were sequenced: they corresponded to the 711 bp full coding sequence and a shorter 594 bp sequence with an internal deletion. Each of the two longer clones contained a single base difference from the consensus of the three, which were corrected by PCR directed mutagenesis.

### RT-PCR analysis

COS-1 cells were transfected with pCMV5-Flag mouse Tgif2, human TGIF2, or mouse/human chimeras. RNA was isolated using an Absolutely RNA kit (Stratagene), and RT-PCR was carried out with Ready To Go PCR beads (Amersham Paharmacia Biotech). Analysis of Tgif2 expression in mouse tissues was carried out by PCR, from first strand cDNAs (Clontech). PCR products were analyzed by agarose gel electrophoresis and southern blot for quantification.

### Luciferase assays

36–40 h after transfection, cells were lysed in Promega passive lysis buffer and assayed for luciferase activity with a Berthold LB 953 luminometer. A pCMVh Renilla luciferase reporter (Promega) was included in all transfections to monitor transfection efficiency. Firefly luciferase was assayed using a luciferase assay kit (Promega) and Renilla luciferase activity was assayed with 0.09 μM coelenterazine (Biosynth) in 25 mM Tris, pH 7.5,100 mM NaCl.

### Immunoprecipitation and western blotting

36 h after transfection, COS-1 cells were lysed by sonication in 100 mM NaCl, 50 mM HEPES, pH 7.8,20% glycerol, 0.1% Tween 20,0.5% NP40 with protease and phosphatase inhibitors. Immunocomplexes were collected on Flag M2-agarose (Sigma). Following SDS-PAGE, proteins were electroblotted to Immobilon-P (Millipore), incubated with antisera specific for: Flag (Sigma), Myc (9E10; Sigma), or Smad2/Smad3 (Upstate), and visualized with horseradish peroxidase-conjugated goat anti-mouse or anti-rabbit Ig (Pierce) and ECL (Amersham Pharmacia Biotech).

## Authors' contributions

TAM participated in the design of the study, performed the cloning, luciferase and interaction assays, and helped to draft the manuscript. DW conceived of the study, participated in its design and RT-PCR analysis. Both authors read and approved the final manuscript.
